# Analysis of innate and acquired resistance to anti-CD20 antibodies in malignant and nonmalignant B cells

**DOI:** 10.7717/peerj.31

**Published:** 2013-02-12

**Authors:** George W. Small, Howard L. McLeod, Kristy L. Richards

**Affiliations:** 1 Lineberger Comprehensive Cancer Center, University of North Carolina at Chapel Hill, NC, USA; 2 Division of Hematology/Oncology, University of North Carolina at Chapel Hill, NC, USA; 3 Department of Pharmacotherapy and Experimental Therapeutics, UNC Eshelman School of Pharmacy, Chapel Hill, NC, USA; 4 UNC Institute for Pharmacogenomics and Individualized Therapy, University of North Carolina at Chapel Hill, NC, USA

**Keywords:** Rituximab, Ofatumumab, CD20 expression, mRNA splice variants, Complement-dependent cytotoxicity, Drug resistance, Lymphoblastoid cell line

## Abstract

The anti-CD20 monoclonal antibody, rituximab, provides a significant therapeutic benefit for patients with B-cell disorders. However, response to therapy varies and relapses are common, so an understanding of both inherited and acquired rituximab resistance is needed. In order to identify mechanisms of inherited resistance, sensitive versus resistant individuals were selected from a survey of 92 immortalized lymphoblastoid B-cell lines from normal individuals. Levels of CD20 protein and surface expression were lower in the resistant group. In contrast, CD20 mRNA levels were not correlated with susceptibility, suggesting regulation at a post-transcriptional level. To examine acquired resistance, resistant sublines were selected from both lymphoblastoid as well as lymphoma cell lines. Confirming previous findings, there was significant down-regulation of CD20 protein expression in all the resistant sublines. CD20 mRNA splice variants are reported to be associated with development of resistance. Three splice variants were observed in our cell lines, each lacking the binding epitope for rituximab, but none were associated with rituximab resistance. The second generation anti-CD20 mAb, ofatumumab, was more active compared with rituximab *in vitro* in the survey of all B-cell lines, mirroring results that have been reported previously with malignant B-cells. These studies show that normal B-lymphoblastoid cell lines can be used to model both innate and acquired mechanisms of resistance. They validate the important role of CD20 expression and enable future genetic studies to identify additional mediators of anti-CD20 mAb resistance.

## Introduction

The therapeutic monoclonal antibody rituximab, directed against the B-cell associated protein CD20, was the first mAb approved for cancer therapy in the United States and has become an integral part of the treatment regimens for B-cell malignancies (Molina, 2008; van Meerten & Hagenbeek, 2009). Rituximab has also been used to treat a variety of autoimmune diseases with B-cell involvement, including rheumatoid arthritis, systemic lupus erythematosus, and multiple sclerosis (Emer & Claire, 2009; Gurcan et al., 2009; Ibrahim, Dimachkie & Shaibani, 2010). Anti-CD20 mAbs are classified as either type I or II. Type I antibodies, such as rituximab, are distinguished by their ability to redistribute CD20 into lipid rafts and induce (CDC) complement-directed cytotoxicity (Cragg et al., 2003). Type II antibodies have reduced CDC capacity. Both type I and II are capable of phagocytosis and (ADCC) antibody-dependent cytotoxicity (Beers et al., 2010; Lim et al., 2010). There is controversy regarding the importance of CDC versus ADCC and even direct cell killing in mediating the clinical efficacy of rituximab (Beers et al., 2010; Lim et al., 2010). The discussion is further complicated by the variability of these effector systems when comparing humans and animal models. However the evidence supporting CDC as an important effector is strong enough to have influenced development of the next generations of anti-CD20 mAbs. A number of 2nd/3rd generation anti-CD20 mAbs have now been developed with either enhanced CDC or ADCC function, with the majority of anti-CD20 mAbs reaching clinical trials being type I mAbs (Lim et al., 2010; Oflazoglu & Audoly, 2010).

Despite widespread treatment successes, resistance is an important issue. For instance, in follicular lymphoma, only ∼50% of patients respond to initial treatment with single-agent rituximab (McLaughlin et al., 1998). Furthermore, the majority of responders eventually become refractory to rituximab (Davis et al., 2000). Therefore, there is a need to understand both the determinants responsible for *de novo* rituximab sensitivity as well as the mechanisms leading to acquired resistance.

Previous reports of cell autonomous factors leading to rituximab resistance have focused on alterations in malignant lymphoma cells, but since anti-CD20 antibodies are used against normal B-cells as well, comparing mechanisms of resistance in these cells is of interest. Also, studying rituximab response in normal B-cells allows inherited, rather than acquired (either during malignant transformation or as a result of therapy) genetic factors to be identified. In this report, variability in anti-CD20 mAb response was assessed using Epstein Barr Virus (EBV)-immortalized human lymphoblastoid cell lines (LCLs) from the Centre d’Etude du Polymorphisme Humain (CEPH). These LCLs from normal individuals as well as established B-cell lymphoma cell lines were then used in a comparative study to examine factors influencing both innate as well as acquired rituximab resistance. The second generation CD20 mAb, ofatumumab, was assayed alongside rituximab to compare the two agents.

In rituximab-naïve B cells, CD20 surface expression is a proven determinant of innate rituximab sensitivity (van Meerten et al., 2006). The levels of expression of complement regulatory proteins, CD55 and CD59, have also been suggested to affect response to rituximab (Golay et al., 2001; Takei et al., 2006). Acquired rituximab resistance in B-cell lymphomas following exposure to rituximab has likewise been associated with reduced levels of CD20 and increases in CD55/59 expression or activity (Czuczman et al., 2008; Ge et al., 2011; Golay et al., 2001; Takei et al., 2006). Using lymphoma cell lines to derive resistant sublines, two recent reports have observed the appearance of a unique protein(s), immunoreactive with CD20 antibody, and associated with acquired rituximab resistance (Czuczman et al., 2008; Henry et al., 2010). This band has been identified as an alternative CD20 mRNA transcript (Henry et al., 2010). Transcription of full-length CD20, encoded by an 8-exon gene, exists as a ∼35 kDa transmembrane phosphoprotein (Tedder et al., 1989). Its exact function is unknown, but binding of rituximab results in increased Ca^2+^ flux, activation of Src family tyrosine kinases, and subsequent reorganization of CD20 into lipid raft domains (Czuczman et al., 2008; Polyak, Tailor & Deans, 1998; Popoff et al., 1998; Semac et al., 2003). The mRNA splice variant associated with rituximab resistance (Henry et al., 2010) results in a truncated protein, eliminating the rituximab epitope but potentially retaining any functions of the inner cytoplasmic tail, which includes potential phosphorylation sites as well as domains necessary for reorganization into lipid rafts (Polyak, Tailor & Deans, 1998; Tedder & Schlossman, 1988).

Based on results achieved with rituximab, subsequent generations of anti-CD20 antibodies are under development. Ofatumumab, a fully humanized anti-CD20 antibody, was recently FDA approved (Lemery et al., 2010). Ofatumumab may have advantages over rituximab in that it dissociates at a slower rate compared to rituximab, and its epitope, which is distinct from that of rituximab, includes a membrane proximal region that may facilitate more potent complement-dependent cytotoxicity (CDC) (Cheson, 2010; Pawluczkowycz et al., 2009; Teeling et al., 2004; Teeling et al., 2006). Here we explore mechanisms of innate and acquired resistance to anti-CD20 mAbs in nonmalignant cells, comparing them with known results in malignant B cells, thus establishing similarities that can be exploited in large-scale screening studies using nonmalignant cells.

## Materials and Methods

### Monoclonal antibodies and human serum

Rituximab (Rituxan™; Genentech, Inc.) and ofatumumab (Arzerra™; GlaxoSmithKline, Inc.) were purchased through the North Carolina Cancer Hospital pharmacy. Pooled human serum was obtained from volunteers and used as a source of complement.

### Cell lines, cell culture, and establishment of resistant lines

The CEPH cell lines, EBV-immortalized lymphoblastoid cell lines from normal individuals, were obtained from the Coriell Institute (Camden, NJ) and lymphoma cell lines were obtained from the Lineberger Comprehensive Cancer Center (LCCC) Tissue Culture Facility (Supplementary Table 1). Cell lines were maintained in RPMI media containing 10% fetal bovine serum (FBS), 100 units/ml penicillin G sodium, and 100 µg/ml streptomycin sulfate except for the Daudi cell lines which were maintained in media containing 20% FBS. For the establishment of resistant cell lines, parental cells were exposed to 10 µg/ml rituximab in the presence of 25% human serum for 24 h. Treated cells were then returned to normal media and allowed to recover for 4–5 days. This process was repeated for approximately 10 passages.

### Microarray gene expression analysis

Publicly available gene expression data for 57 of the lymphoblastoid CEPH cell lines used in this study were available in Gene Expression Omnibus (GEO) Dataset GSE12626 (time = 0 samples were used). CEL files were downloaded from the GEO site and normalized using Robust Multichip Average (RMA) in the Bioconductor package using R statistical software, version 2.12.1. Expression data from the two probesets (210356_x_at, 210356_x_at) interrogating *MS4A1* (CD20) were averaged. Average expression was plotted against viability of each cell line in rituximab. 

### Proliferation assays

Cells were seeded at 5 × 10^4^ cells in a 96-well plate in a total volume of 100 µl media containing 25% pooled human serum with or without 10 mg/ml rituximab overnight. Proliferation was then measured using Alamar Blue (AbD Serotec Ltd.) according to manufacturer’s instructions. Dye was added to samples and incubated for approximately eight hours. Fluorescence was then measured with excitation at 535 nm and emission at 595 nm. Proliferation was expressed relative to samples without antibody (rituximab or ofatumumab).

### RNA isolation and PCR

Total cellular RNA was extracted using TRIzol (Invitrogen) and RNA (1 µg) reverse-transcribed into cDNA using Transcriptor First Strand cDNA Synthesis kit (Roche) according to manufacturer instructions. Conventional PCR was performed using Taq PCR Master Mix (Qiagen Inc.) and quantitative PCR (qPCR) was performed using SsoFast™ EvaGreen^®^ (Bio-Rad) master mix. Reactions used 400 nM each of forward and reverse primers prepared by the Nucleic Acids Core Facility (University of North Carolina at Chapel Hill). The annealing temperature was 59 °C. The primers used were: 1-CD20F (5’-GAGCCAATGAAAGGCCCTATT-3’) and CD20R (5’-TGCTGCCAGGAGTGATCCGGAA-3’) for analysis of CD20 mRNA; 1-CD20F and 350-CD20R (5’-AGCTATACAAGTTCCAGTG-3’) for specific analysis of the 350 bp splice variant; 1-CD20F and 650-CD20R1 (5’-CACTGACAAAATGCCCAAACACTTC-3’) or 650-CD20R2 (5’-GCTATTACAAGTTCCAAACACTTCC-3’) for specific analysis of the 650 bp splice variants. To obtain sequence data, PCR products were extracted from gel slices using a QIAquick Gel Extraction kit (Qiagen) and sequenced using the PCR primers (Genomic Analysis Core Facility, University of North Carolina).

### Western blotting

2 × 10^6^ cells were lysed in RIPA buffer (Dulbecco’s PBS, 0.1% Sodium Dodecyl Sulfate, 0.5% Sodium Deoxycholate, and 1% Igepal NP-40 supplemented with 1 mM PMSF, 1x cOmplete protease inhibitor cocktail (Roche Diagnostics, Indianapolis, IN), and 1x Phosphatase Inhibitor Cocktail 2 (Sigma, St. Louis, MO). An aliquot was used for protein determination using the BCA protein assay kit (Pierce Technology, Inc., St. Louis, MO). The remaining sample was mixed with 0.5 vol. of 3x SDS sample buffer (1x equals 31.25 mM Tris-HCl (pH 6.8), 2% SDS, 5% 2-mercaptoethanol, 10% sucrose, and 0.005% bromophenol blue) and heated for 5 min at 95 °C. Equivalent protein amounts were run on SDS-PAGE in Tris/glycine/SDS buffer using 10% acrylamide gels. Resolved proteins were then transferred to PVDF membranes. Blots were blocked using a 5% solution of nonfat dry milk in 20mM Tris-HCl, pH7.4, 140 mM NaCl, 0.1% Tween 20 and exposed to antibodies in the same buffer. A rabbit anti-CD20 antibody (Thermo Scientific, Cat. # RB-9013) was used. Anti-LMP-1 was a kind gift from Dr. Blossom Damania (Lineberger Comprehensive Cancer Center, University of North Carolina). Anti-β-actin (Sigma) was used as a load control. Horseradish peroxidase-conjugated secondary antibodies were used with ECL detection reagent (GE Healthcare Bio-Sciences Corp.) to visualize immunoreactive bands. Protein levels were quantified with an Agfa Duoscan T2500 scanner (Agfa Corp.) followed by densitometry using NIH Image 1.61.

### Flow cytometry

1.0 × 10^6^ cells were centrifuged, then resuspended in 100 µl cold Dulbecco’s PBS buffer containing 0.1% sodium azide, 1.0% FBS, and 1% pooled human serum and kept on ice for 30 min. For analysis of CD20 expression, 10 µl of FITC-αCD20 (BD Biosciences) was added and samples incubated for an additional 45 min before adding an equal volume of buffer. Samples were centrifuged and pellets resuspended in 0.5 ml of the same buffer containing 5 nM TOPRO-3 (Invitrogen), which was used to distinguish viable cells. For analysis of CD55 and CD59, 10 µl of premixed R-PE-αCD55/FITC-αCD59 (Invitrogen) antibodies were used per sample. Phenotyping was performed on a Beckman-Coulter (Dako) CyAn flow cytometer. Data analysis was performed with FlowJo software (Tree Star, Inc.).

### Statistical analyses

Unpaired, two-tailed *t* tests were done using Prism software (version 2.0; GraphPad Software). Correlation coefficients were calculated with Stata (version 10.1) or Microsoft Excel.

## Results

### Anti-CD20 antibodies induce cell death in lymphoblastoid and lymphoma cells in the presence of complement

**Figure 1:**
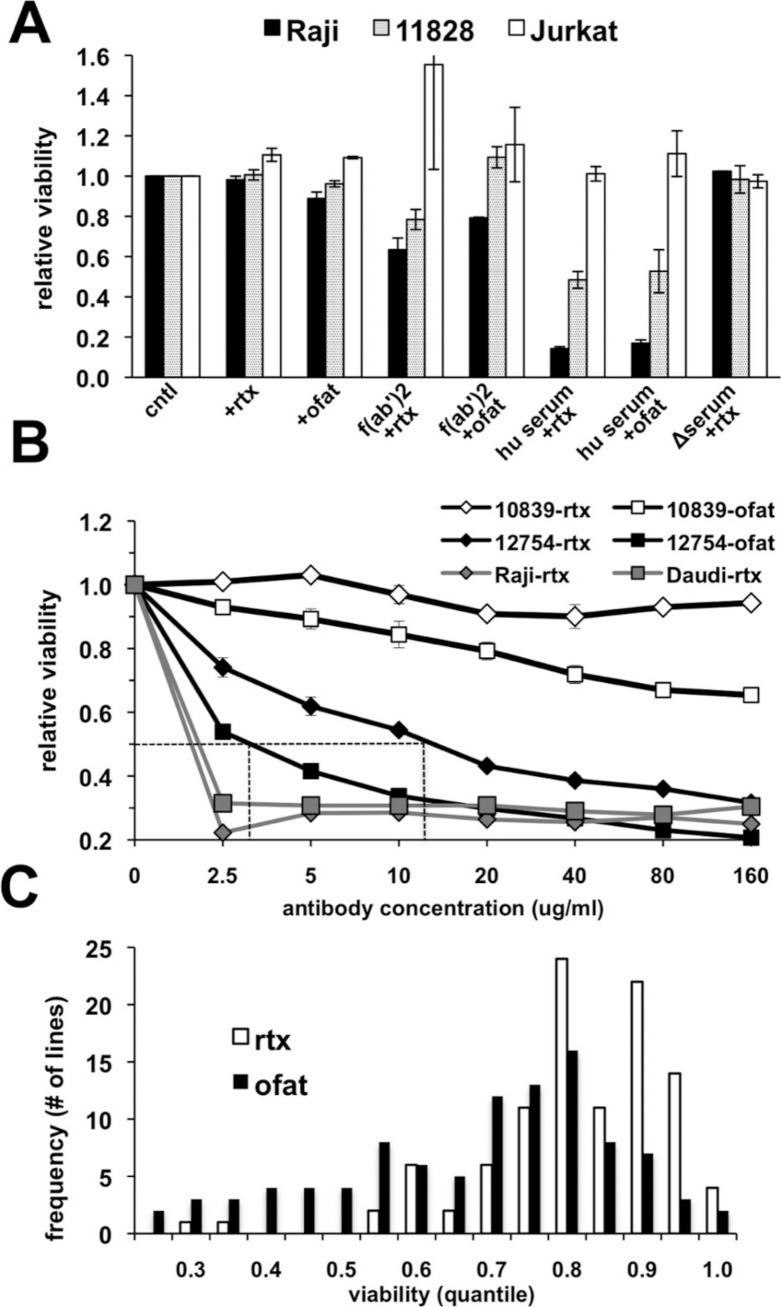
Variable cell responses in anti-CD20 mediated CDC assays. (A) Cells were treated with 10 µg/ml of rituximab or ofatumumab under various conditions: in normal media, in the presence of: −F(ab’)_2_, −25% human serum (hu serum), or -heat-inactivated serum (Δserum) overnight. Cell viability was then quantitated using Alamar Blue. Results are represented as a ratio of viability under each condition: with/without antibody. Results are expressed as an average of a single experiment with duplicates ±SEM and are representative of three such experiments. (B) To establish dose response curves, cells were treated with serially diluted concentrations of antibody in the presence of 25% human serum. Results are expressed as indicated above and representative of at least three experiments. (C) Survey of CEPH LCLs sensitivity toward rituximab and ofatumumab. A panel of CEPH cell lines (*n* = 92) were assayed for their relative sensitivity toward anti-CD20 antibodies using CDC assays containing 10 µg/ml of either rituximab or ofatumumab in the presence of 25% human serum. The results are plotted as a frequency histogram showing the distribution of responses to either antibody. Data used are the average of at least 5 individual experiments performed in duplicate.

The suitability of an *in vitro* assay for high throughput screening of cell lines for anti-CD20 monoclonal antibody (mAb) sensitivity was first tested using CD20-positive lymphoma (Raji) and lymphoblastoid (GM 11828) cell lines as well as a CD20-negative (Jurkat) T-cell line. The response of these cells following treatment for 18 h with antibody alone or in the presence of either human serum, heat-inactivated human serum, or F(ab’)_2_ fragments was quantitated using AlamarBlue as an indicator for metabolic activity of the remaining viable cells. Rituximab alone had no observable effect under these conditions (Fig. 1A). A small decrease in viability could be noted if the treatment time was extended to 48–72 h (data not shown). A minimal response was observed with ofatumumab in both CD20+ cell lines with a decline in viability of only ∼10% in Raji cells (Fig. 1A). Since anti-CD20 antibody alone had only a minimal effect on cell viability, we tested several previously published methods to increase cell killing *in vitro* after rituximab treatment. Cross-linking of CD20 through antibody binding is reported to induce apoptosis (Cardarelli et al., 2002; Ghetie, Bright & Vitetta, 2001; Shan, Ledbetter & Press, 1998), and indeed, cells were more sensitive when treated with rituximab in the presence of anti-human F(ab’)_2_ fragments. However, inclusion of human serum as a complement source resulted in the greatest declines in CD20^+^ cell viability, which was reduced by ∼85% in Raji cells treated with either antibody (Fig. 1A). This decline was observed only in CD20-positive cells; the CD20-negative Jurkat cell line was unaffected. Heat inactivation effectively prevented cell death from occurring, consistent with complement dependent activity.

The effectiveness of rituximab and ofatumumab were compared in dose response assays. Ofatumumab was approximately four-fold more potent at cell killing, comparing the IC_50_ between the two antibodies in sensitive cell lines (Fig. 1B). In more resistant cell lines, a dose effect and increased activity at a lower concentration could frequently be observed for ofatumumab when none was apparent for rituximab (Figs. 1B and 1C). The lymphoma lines, Daudi and Raji were quite sensitive to both anti-CD20 antibodies relative to the lymphoblastoid lines, with maximal cell death occurring at 2.5 µg/ml, the lowest antibody concentration tested (Fig. 1B).

### Response to anti-CD20 antibodies is variable among normal LCLs, but degree of rituximab and ofatumumab response is correlated

Ninety-two CEPH LCLs as well as eight lymphoma cell lines were then surveyed *in vitro* to assess relative sensitivity to rituximab and ofatumumab. Ten µg/ml of anti-CD20 mAb was chosen since this concentration is commonly used for *in vitro* rituximab assays (Czuczman et al., 2008; Di Gaetano et al., 2003; Golay et al., 2001), and approximates the IC_50_ for rituximab in sensitive LCLs (Fig. 1B). Results were averaged from at least five separate experiments. LCLs yielded a broad spectrum of response to rituximab with a more negatively skewed distribution for ofatumumab in comparison to rituximab, reflecting its greater potency (Fig. 1C). LCLs typically showed more sensitivity to ofatumumab, but the correlation between the two anti-CD20 mAbs was relatively strong (Pearson’s *r* = 0.795, Spearman’s ρ = 0.770, Supplementary Fig. 1A). To rule out the possibility that variability in EBV levels was affecting rituximab response, LMP protein expression was examined and did not vary significantly between resistant and sensitive cell lines (Supplementary Fig. 2). The lymphoma cell lines were generally more sensitive to anti-CD20 mAbs, but they likewise displayed a range of response with Daudi and Raji being the most sensitive and DB and SUDHL-10 most resistant (Table 1).

**Table 1:** Comparison of rituximab sensitivity with CD20, −55, −59 surface expression in lymphoblastoid and lymphoma cell lines.

Cell line	Viability^a^	CD20^b^	CD55^b^	CD59^b^
	rituximab, (*n* = 10)	LCLs (*n* = 4)	(*n* = 1)	(*n* = 1)
		Lymphomas (*n* = 2)		
12622	0.28 ± 0.028	53 ± 8.4	205	134
11828	0.31 ± 0.004	28 ± 3.7	130	221
12621	0.32 ± 0.004	41 ± 9.8	254	185
12828	0.37 ± 0.031	22 ± 3.0	232	229
12754	0.64 ± 0.064	34 ± 4.5	48	273
				
12803	0.88 ± 0.009	20 ± 2.6	210	189
12698	0.93 ± 0.019	8 ± 0.1	104	170
11988	0.90 ± 0.036	13 ± 3.2	167	244
11989	1.00 ± 0.012	24 ± 5.1	114	119
10839	1.03 ± 0.013	19 ± 2.6	217	207
Daudi	0.17 ± 0.028	85 ± 15.0	57	175
Raji	0.24 ± 0.052	50 ± 5.0	38	61
BJAB	0.49 ± 0.089	77 ± 1.8	19	8
SUDHL4	0.28 ± 0.023	67 ± 50	29	16
HT	0.57 ± 0.045	81 ± 1.0	198	382
Farage	0.74 ± 0.039	131 ± 78	21	69
DB	0.77 ± 0.021	118 ± 10	29	44
SUDHL10	0.83 ± 0.036	62 ± 53	19	7

**Notes.**

a Viability is represented as a ratio of growth with/without 10 µg/ml of rituximab in the presence of 25% human serum and expressed as an average ±SEM. Cell lines are grouped as: CEPH lines or lymphoma lines and listed from most sensitive to most resistant based on results from viability assays conducted in the presence of rituximab and human serum. Median viability of LCLs was 0.77 and the bold line separates “sensitive” (above) and “resistant” (below) LCLs.

b CD −20, −55, and −59 surface expression is represented as relative median fluorescence.

**Figure 2:**
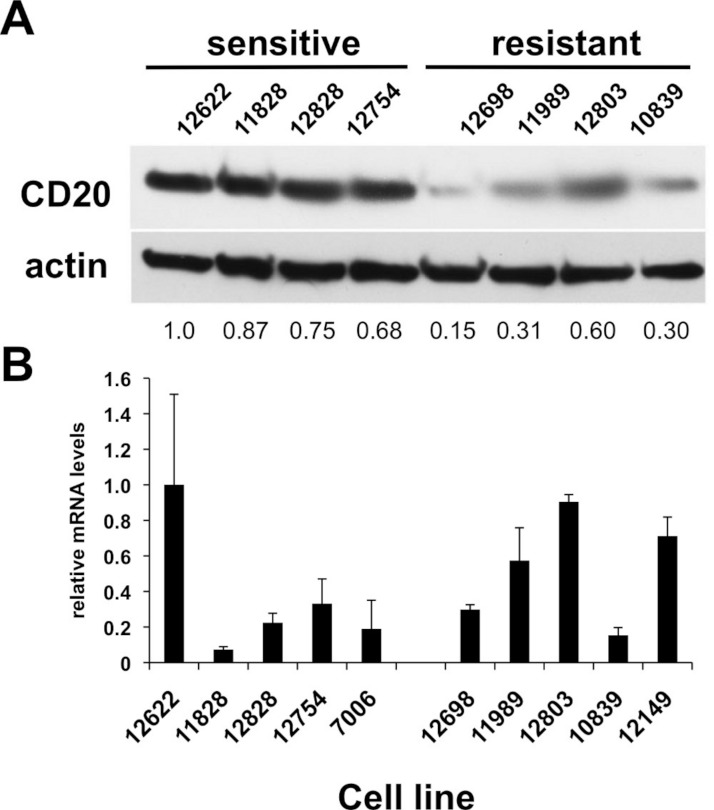
CD20 protein and mRNA levels in sensitive or resistant lymphoblastoid cell lines. A) Western blotting was performed on cellular extracts from lymphoblastoid cell lines previously identified in surveys of cell lines as either sensitive (lanes 1–4) or resistant (lanes 5–8) toward rituximab. Following densitometry, relative CD20 protein levels are expressed by normalizing to the leftmost sample (12622) after correction for equal protein loading using β-actin as a load control. B) CD20 mRNA transcript levels were measured by qPCR. GAPDH was used as a load control and data is expressed by normalizing to the leftmost sample (12622). Results are the average of duplicates (±SEM) and representative of three experiments.

### CD20 surface expression, but not mRNA expression, predicts rituximab sensitivity in LCLs

**Figure 3:**
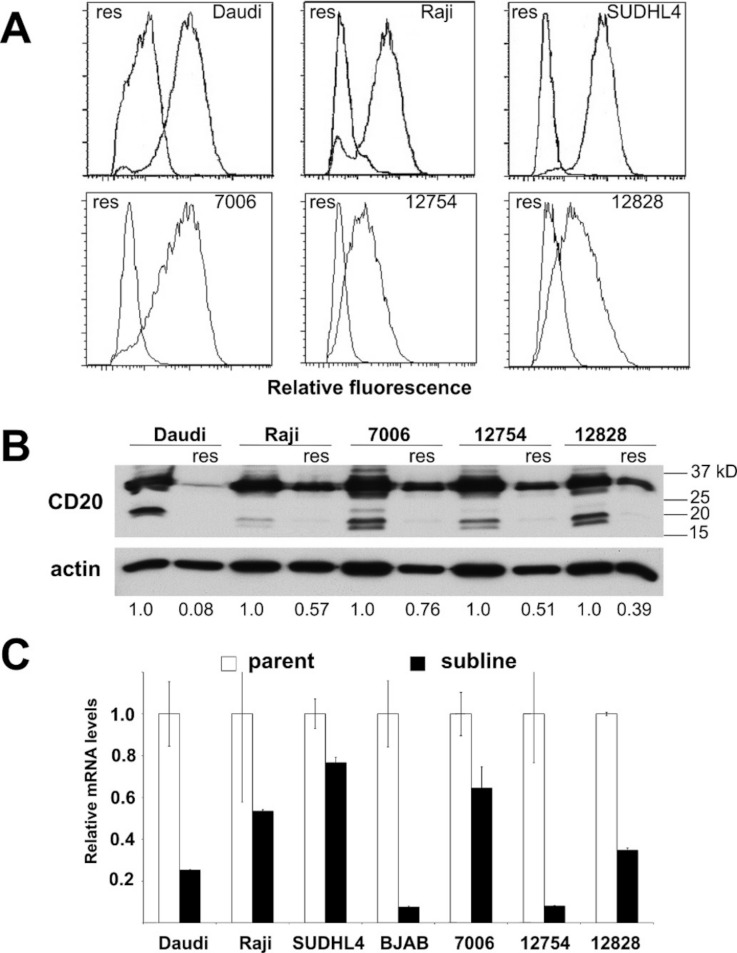
CD20 surface expression as well as protein and mRNA levels in parental and resistant sublines. A) Histogram of CD20 surface expression following flow cytometric analysis of parental cell lines (right trace, each panel) and resistant sublines (left trace). B) Western blot of CD20 protein expression (upper band). CD20 protein levels are expressed by normalizing to the corresponding parental line after correction for equal protein loading using β-actin as a load control. C) Relative CD20 mRNA levels were estimated using qPCR. GAPDH was used as a load control and data are normalized to the parental line. Results are the average of duplicates (±SEM) and representative of three experiments.

Of the LCLs surveyed, five inherently sensitive and five inherently resistant lines (i.e. cell lines below and above the median viability of LCLs in rituximab, respectively) were selected for further characterization. Relative levels of CD20 surface expression were examined by flow cytometry (Table 1). A plot of CD20 surface expression versus viability among the LCLs demonstrated a moderately strong correlation between CD20 surface expression and sensitivity to rituximab (*R*^2^ = 0.52). The lymphoma lines generally exhibited higher levels of CD20 surface expression and greater rituximab sensitivity than the lymphoblastoid lines. However, some lymphoma lines had even higher CD20 expression than Raji and Daudi cells, yet were less sensitive to rituximab (Table 1, Fig. 3A), suggesting that there are other contributing factors in addition to CD20 levels.

The complement inhibitors, CD55 and CD59 have been suggested to regulate susceptibility to rituximab and complement (Golay et al., 2001; Takei et al., 2006). Surface expression by flow cytometry (Table 1) showed no correlation between sensitivity and CD55 or CD59 expression in either LCLs (*R*^2^ = 0.054, *R*^2^ = 0.013 respectively) or in the lymphoma cell lines (*R*^2^ = 0.032, *R*^2^ = 0.014). It remains possible that changes in the distribution of CD55/59 relative to CD20 rather than total protein amount could impact rituximab sensitivity. However, the only association observed in this experiment was between the expression of CD55 versus CD59 in the lymphoma cells lines, perhaps reflecting their coordinated activity in complement inhibition (Table 1, *R*^2^ = 0.94).

CD20 surface expression corresponded to CD20 total protein expression detected by Western blot analysis (Table 1, Fig. 2A). The average protein level of four of the sensitive LCL cell lines, 0.83 ± 0.070, was significantly higher than in resistant lines, 0.34 ± 0.094 (*p* < 0.006, Fig. 2A). In contrast, no significant difference in CD20 (*MS4A1*) mRNA levels was observed. Sensitive lines averaged 0.37 ± 0.140 relative to resistant lines that averaged 0.54 ± 0.098 (*n* = 10, *p* < 0.34) (Fig. 2B). This observation was consistent with results from gene expression array analysis. Publicly available gene expression data for 57 of the lymphoblastoid CEPH cell lines (Gene Expression Omnibus Dataset GSE12626) was used to compare CD20 (*MS4A1*) mRNA expression to viability in rituximab. There was no correlation between CD20 mRNA levels and viability in rituximab (*R*^2^ = 0.01, Supplementary Fig. 1B).

### Acquired rituximab resistance is also associated with reduced CD20 levels

Periodic exposure of cell lines to rituximab followed by recovery led to the development of resistant sublines (Supplementary Fig. 3A). Four out of four lymphoma lines and three out of four lymphoblastoid lines yielded resistant sublines. All of the rituximab resistant lines were also resistant to ofatumumab (data not shown). In order to examine the stability of the resistant phenotype among the different cell lines, a separate culture of each of the resistant sublines was grown in the absence of selection. In contrast to other reports (Czuczman et al., 2008), the resistant phenotype appeared to be at least partly reversible under our conditions. At two months, there was partial loss of the resistant phenotype in the LCLs. Resistance was retained in three of the four lymphoma sublines at two months, but by six months, three of the four lymphoma sublines had regained some sensitivity to rituximab (Supplementary Fig. 3B). It has been proposed that acquired rituximab resistance may occur through epigenetic modification (Sugimoto et al., 2009). However, attempts to hasten re-expression using epigenetic modifiers (trichostatin A as a histone deacetylase inhibitor and 5-azacytidine as a DNA methyltransferase inhibitor) were not successful (data not shown).

Acquired resistance was accompanied by down-regulation of CD20 expression in all the sublines relative to their respective parental lines as evidenced by decreased surface expression (Fig. 3A). Decreases in surface expression corresponded to lower total protein levels as determined by Western blot (Fig. 3B). Likewise, reductions in CD20 mRNA levels were consistently seen in all sublines as well, indicating regulation of CD20 expression at the transcriptional level in the case of acquired rituximab resistance (Fig. 3C). These results were distinct from earlier results obtained using LCLs with an innate rituximab resistance, where mRNA levels were not correlated with resistance (Fig. 2).

### Alternative splicing of CD20 mRNA is not associated with rituximab resistance in LCLs

**Figure 4:**
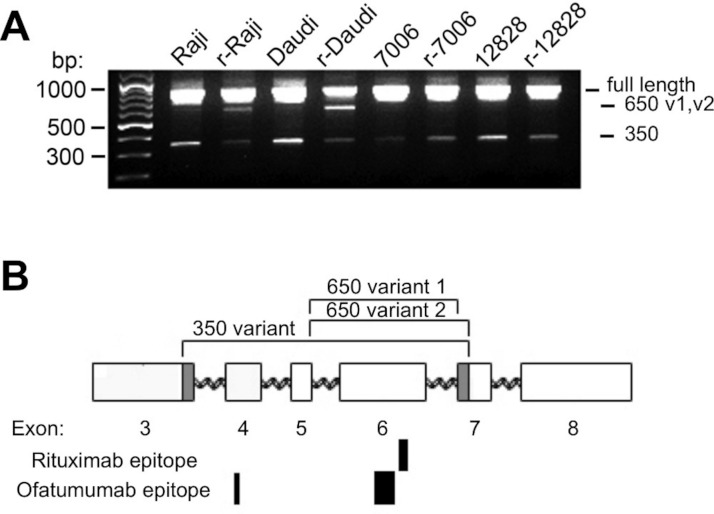
CD20 mRNA splice variants. A) Agarose gel electrophoresis of PCR products from parental and resistant sublines following amplification of CD20 mRNA transcripts. Primers near the start and end of CD20 were used in order to look for potential splice variants. B) Schematic diagram of the CD20 gene. Exons are represented by rectangles. Brackets indicate regions removed in the splice variants. The shaded region in exons 3 and 7 shows the location of non-standard splice sites. The relative size and positions of the rituximab and ofatumumab epitopes are represented by the solid rectangles below.

Alternative splicing of the CD20 gene (*MS4A1*) results in three mRNA transcript isoforms which encode the same full-length protein (Tedder et al., 1989), and recent studies show evidence of truncated variants associated with rituximab resistance (Czuczman et al., 2008; Henry et al., 2010; Terui et al., 2009). To explore the possibility that acquisition of rituximab resistance may be accompanied by the induction of alternate CD20 mRNA splice variants, cDNA from both parental and resistant sublines was amplified. In addition to the primary full-length transcript of approximately 900 bp (Fig. 4A), a minor ∼350 bp product was seen all cell lines. Sequence analysis confirmed that this band was the previously described ΔCD20 splice variant lacking an internal 501 bp fragment spanning from mid-exon 3 to mid-exon 7 (Henry et al., 2010). The resultant protein has a predicted size of 15kD and lacks the 4 transmembrane domains as well as the extracellular loops serving as antigenic sites.

In addition to these products, a third ∼650 bp PCR band could be observed, more prominent in the lymphoma lines than in the LCLs (Fig. 4A). The ∼650 bp product from r-Daudi was subcloned into a sequencing vector and analyses were performed on individual colonies. Sequencing revealed two species of a previously unidentified splice variant with alternative 3’ acceptor sites. The alternate splice junction of species 1 joins exon 5 to exon 7 forming an in-frame mRNA transcript of 660 bp (Fig. 4B). The alternate splice junction of species 2 likewise occurs at the 3’ end of exon 5 but is instead spliced at the alternative splice site 39 bp downstream from the 5’ end of exon 7 resulting in a 621-bp in-frame transcript (Fig. 4B). The resulting proteins have a predicted size of 24 kD, retaining 3 transmembrane domains but lacking the external antigenic sites and one transmembrane domain.

Western blots probed with an antibody directed against the carboxy terminus of CD20, common to all transcripts, showed global down-regulation of all species of the CD20 protein in the resistant sublines (Fig. 3B). The primary, immunoreactive band migrated as a doublet at ∼33/35 kD, presumably including the phosphorylated form(s) of full-length CD20. Increased exposure of the western blot also revealed a doublet at ∼17/19 kD which could be assigned to the 350 bp splice variant seen in agarose gels (Fig. 4A). It likewise decreased with the acquisition of a resistant phenotype making it an unlikely effector of rituximab resistance, in contrast to a previous report (Henry et al., 2010). A third, intermediate band could be observed at approximately 27 kD and was attributed to the 650 bp splice variant (Fig. 4B). However, the 27 kD band likewise decreased with the acquisition of resistance.

### Global down-regulation of CD20 mRNA species with acquisition of resistance

**Figure 5:**
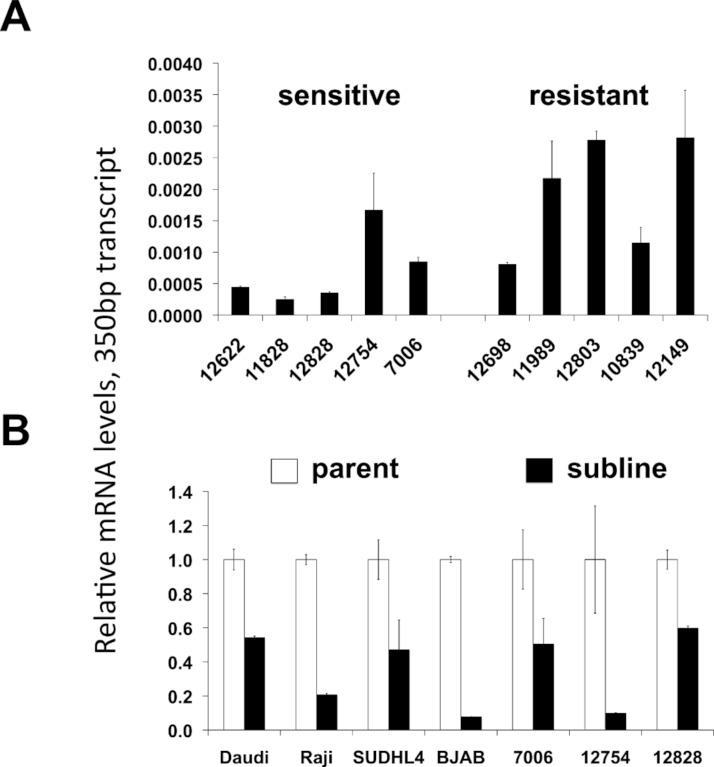
CD20 mRNA levels of the 350 bp splice variant. Levels of the 350 bp CD20 mRNAs isoform are shown in (A) lymphoblastoid lines that are relatively sensitive or resistant to rituximab, and (B) parental and resistant sublines. Relative CD20 mRNA levels were estimated by qPCR. GAPDH was used as a load control and data are normalized to the level of full-length CD20 of the corresponding parental line, (Fig. 2B). Results are the average of duplicates (±SEM).

A potential increase in the 650 bp variants in the resistant sublines seen by standard PCR (Fig. 4A) was not confirmed by qPCR. There was no statistically significant difference in the mRNA levels of the ∼650 bp splice variant species when comparing innately resistant to sensitive LCLs (Supplementary Fig. 4A). The two CD20 mRNA ∼650 bp splice variant species were roughly in proportion to each other (data not shown). The 350 bp splice variant was increased in the innately resistant lines in comparison to the sensitive cell lines (Fig. 5A, *p* < 0.005). However, the levels of the 350 bp splice variant were approximately 350 × less than levels of full length CD20 mRNA, making its ability to contribute to a resistant phenotype uncertain. Comparing sensitive parental cell lines with resistant sublines, both the ∼650 bp and 350 bp variants paralleled levels of full-length CD20 mRNA, decreasing with the acquisition of a resistant phenotype (Figs. 3C, 5B, Supplementary Fig. 4B). Also, the lymphoma lines also appear to generally express more of the splice variant mRNAs relative to the LCLs (Fig. 5B), consistent with previous reports (Grosso, Martins & Carmo-Fonseca, 2008). In summary, although splice variants could be detected, they did not appear to be associated with acquired resistance.

## Discussion

The outstanding clinical success of rituximab has provoked a search for even more effective anti-CD20 antibodies, as well as a better understanding of resistance mechanisms to guide development and optimize the use of these agents clinically. Anti-CD20 antibodies are used clinically to eradicate both malignant and nonmalignant B-cells (e.g. in the treatment of autoimmune diseases), so understanding inherited factors that affect both malignant and nonmalignant B-cells is an important component of this work. For both malignant and nonmalignant B-cell disorders, understanding both inherited (germline) and acquired (somatic) resistance mechanisms is essential. This report explores for the first time a large number of nonmalignant lymphoblastoid B-cell cell lines to explore *innate* variability in sensitivity to anti-CD20 antibodies. We have confirmed that LCLs can be killed by CDC *in vitro*, and furthermore discovered that there is a significant amount of reproducible variability among individual cell lines, implying that innate mechanisms of resistance may play a role in B-cell sensitivity to anti-CD20 antibodies clinically. The variability in response between individuals enables the future search for genetic components of anti-CD20 antibody sensitivity with the use of high-throughput *in vitro* screens in large numbers of individuals, which is currently ongoing in our laboratory (Peters et al., 2011).

Our results confirm that CD20 expression level is an important component of response to anti-CD20 agents. This has been shown repeatedly with malignant B-cells (Bellosillo et al., 2001; Czuczman et al., 2008; Golay et al., 2001), but is less well established with nonmalignant B-cells. Furthermore, we found that control of CD20 expression occurs at a post-transcriptional level in the case of innate resistance of LCLs, in contrast with acquired resistance, which is correlated with CD20 mRNA levels, suggesting transcriptional-level control. Another difference between innate and acquired resistance is that the acquired resistance is not stable in the absence of selection, whereas the innate sensitivity to rituximab is stable over time. This implies that acquired and innate resistance mechanisms are not identical, and warrant separate consideration. Antigenic modulation following exposure to antibody has been suggested to contribute to rituximab resistance. It has been proposed that in the presence of THP-1 monocytes, rituximab/CD20 complexes are shaved from target B cells in a reaction mediated by FCγR with no evidence of CD20 internalization taking place in the target cells (Beum et al., 2006). In contrast, others have reported that CD20 internalization can indeed play a significant role while shaving does not (Beers et al., 2010). Our system, without effector cells, cannot settle this controversy, but experiments are in progress to look at CD20 internalization in our rituixmab resistant cells *in vitro*. In addition, since CD20 levels do not fully account for differences in rituximab resistance, discovery of other novel genes involved in this process is possible.

Our results also reveal the presence of a novel CD20 splice variant (27 kD), as well as a previously reported variant (15–17 kD). The shorter splice variant was noted in transformed LCLs, and was previously reported to be increased, both *in vitro* and *in vivo*, in rituximab-resistant lymphoma cells (Henry et al., 2010). We did not observe the same increase in either lymphoma cell lines or LCLs with acquired resistance, and the ratio of the shorter splice variants to the total amount of CD20 (both mRNA and protein) remained constant or decreased. Furthermore, the splice variants accounted for no more than 5% of the CD20 transcripts in each of the resistant lines. CD20 does form homo- or hetero-oligomeric complexes (Polyak et al., 2008). Therefore, it remains possible that a minor population of ΔCD20 splice variants could still affect the function of CD20 oligomeric complexes or susceptibility to CDC, leaving the role of either splice variant in rituximab resistance to be established.

Although many comparisons in malignant cells have been reported, few reports have compared the efficacy of rituximab and ofatumumab in normal LCLs (Li et al., 2008; Pawluczkowycz et al., 2009). We found no major distinctions, other than the previously reported increased potency of ofatumumab for CDC-mediated cell death. In both innate and acquired resistance, ofatumumab sensitivity closely paralleled rituximab sensitivity, suggesting shared mechanisms *in vitro*. In no case did we find a cell line with discordance between rituximab and ofatumumab sensitivity (either acquired or innate). Still, since the correlation between the two phenotypes was not perfect (*R*^2^ = 0.63), there may be more subtle differences between the two antibodies, or differences that are more apparent *in vivo*, with a full immune system in play.

We note several limitations to our *in vitro* system. First, in the absence of effector cells, at least one component of *in vivo* cell killing, antibody-dependent cell mediated cytotoxicity (ADCC), is not measured. This simplifies our system, allowing high throughput assays to be easily performed, but results must be validated in future clinical studies to calibrate the significance in patients. The contributions of CDC and ADCC remain a debated topic. It is likely that both activities are occurring to some degree, perhaps varying over time. These effector systems may of course also vary within the different environments in vivo (i.e. peripheral blood versus tissue or interstitial spaces). Furthermore, there have been recent reports that allelic variants of both Fc gamma IIIa receptor and of C1q impact anti-CD20 therapy (Racila et al., 2008; Weng & Levy, 2003). Given these considerations, the array of anti-CD20 mAbs now available with enhanced CDC or ADCC function may offer an expanded potential for personalized as well as sequentially-based therapies.

The LCLs used in our experiments are immortalized with EBV, which could confound assumptions about “innate” sensitivity if EBV immortalization somehow affects rituximab sensitivity. However, our unpublished results indicate heritability of rituximab sensitivity segregating in families (*h*^2^ = 0.294), and EBV LMP levels are equivalent in sensitive and resistant lines (Supplementary Fig. 2), so we find this unlikely to be a hindrance. Still, future validation of our findings in non-EBV immortalized cells will be essential. These potential limitations are likely to be outweighed by the capacity now, with the findings from this work, for the high-throughput study of a large number of nonmalignant B-cells for large-scale studies as has been done with other cytotoxic agents (Hartford et al., 2009; Huang et al., 2011; Peters et al., 2011; Watson et al., 2011). The ability to detect and compare both innate and acquired resistance to anti-CD20 antibodies *in vitro* in nonmalignant immortalized B-cells now provides a powerful new reagent for high-throughput genetic studies to further define and delineate the factors underlying both rituximab and ofatumumab resistance.

## Supplemental Information

10.7717/peerj.31/supp-1 Supplementary Fig. 1 Correlation of the sensitivity toward rituximab against ofatumumab sensitivity or CD20 mRNA expression A) Scatterplot of CEPH LCLs sensitivity toward rituximab versus ofatumumab. A panel of CEPH cell lines were assayed for their relative sensitivity toward anti-CD20 antibodies using CDC assays as previously described. Sensitivity is represented as relative viability. Data used are the average of at least 5 individual experiments performed in duplicate. B) Scatterplot of rituximab sensitivity versus CD20 gene expression. Rituximab sensitivity is plotted against publicly available gene expression data for 57 of the lymphoblastoid CEPH cell lines used in this study, available from the Gene Expression Omnibus (GEO) Dataset GSE12626 and using an average of expression data from the two *MS4A1* (CD20) probesets (210356_x_at, 210356_x_at).

10.7717/peerj.31/supp-2 Supplementary Fig. 2 LMP-1 protein levels in sensitive or resistant lymphoblastoid cell lines Western blotting was performed on cellular extracts from lymphoblastoid cell lines previously identified in surveys of cell lines as either sensitive (lanes 1-4) or resistant (lanes 5-8) toward rituximab. Following densitometry, relative LMP-1 protein levels are expressed after correction for equal protein loading using b-actin as a load control. The average (SEM) is provided for the sensitive and resistant groups.

10.7717/peerj.31/supp-3 Supplementary Fig. 3 Characterization of rituximab resistance in sublines Resistant sublines were selected from parental cells as described in Methods. A) Viability assay comparing parental cell line and sublines toward rituximab in presence of human serum. Data are expressed as the average of duplicates (± SEM) and representative of three experiments. B) Viability assay comparing parental cell line, resistant subline, and resistant sublines removed from selective pressure for the indicated times. Data are expressed as the average of two experiments performed in duplicate (± SEM).

10.7717/peerj.31/supp-4 Supplementary Fig. 4 CD20 mRNA levels of the 650bp splice variant Levels of the 650 bp CD20 mRNA isoform (variant 1) measured by qPCR are shown in (A) lymphoblastoid lines that were identified as either relatively sensitive or resistant to rituximab, and (B) parental and resistant sublines. In (A), ratios are relative to the level of full-length CD20 of the lymphoblastoid line, 12622. In (B) resistant sublines are each normalized to the level of 650-bp transcript levels of the corresponding parental line (set to 1). Ratios of the 650 bp transcript to full-length CD20 ranged from 0.0006 to 0.016 in the lymphoblastoid cell lines and from 0.008 to 0.174 in the lymphoma cell lines. Results are the average of duplicates (± SEM).

10.7717/peerj.31/supp-5 Supplementary Table 1 Cell lines used in this study
