# Supplementary figures and images for: Analysis of innate and acquired resistance to anti-CD20 antibodies in malignant and nonmalignant B cells
Source: PeerJ. 2013 Feb 12;1:e31. doi: 10.7717/peerj.31 (PMC3628892; doi:10.7717/peerj.31)

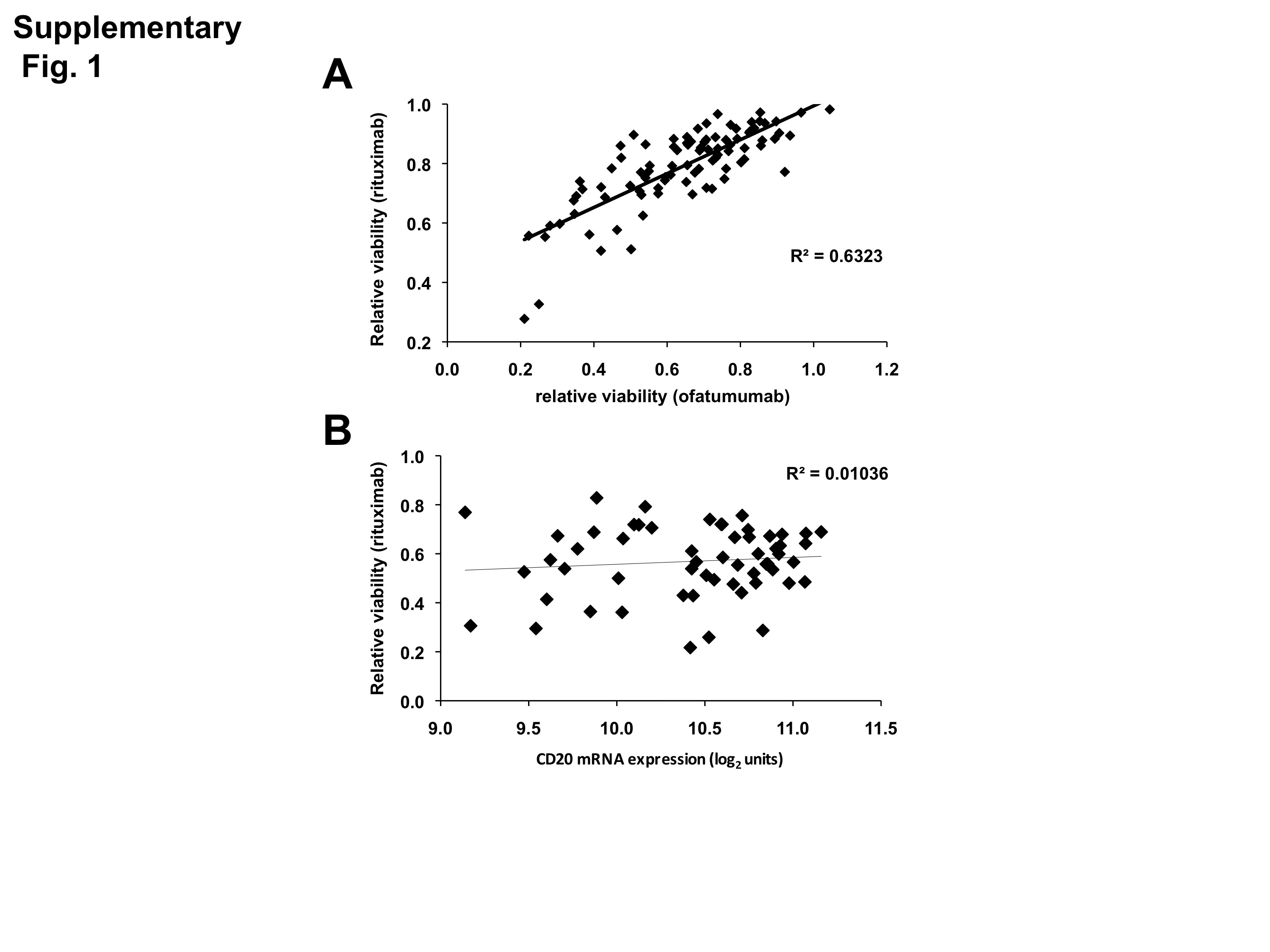

Supplement: Supplementary Fig. 1 — A) Scatterplot of CEPH LCLs sensitivity toward rituximab versus ofatumumab. A panel of CEPH cell lines were assayed for their relative sensitivity toward anti-CD20 antibodies using CDC assays as previously described. Sensitivity is represented as relative viability. Data used are the average of at least 5 individual experiments performed in duplicate. B) Scatterplot of rituximab sensitivity versus CD20 gene expression. Rituximab sensitivity is plotted against publicly available gene expression data for 57 of the lymphoblastoid CEPH cell lines used in this study, available from the Gene Expression Omnibus (GEO) Dataset GSE12626 and using an average of expression data from the two MS4A1 (CD20) probesets (210356_x_at, 210356_x_at). [file peerj-01-31-s001.jpg]

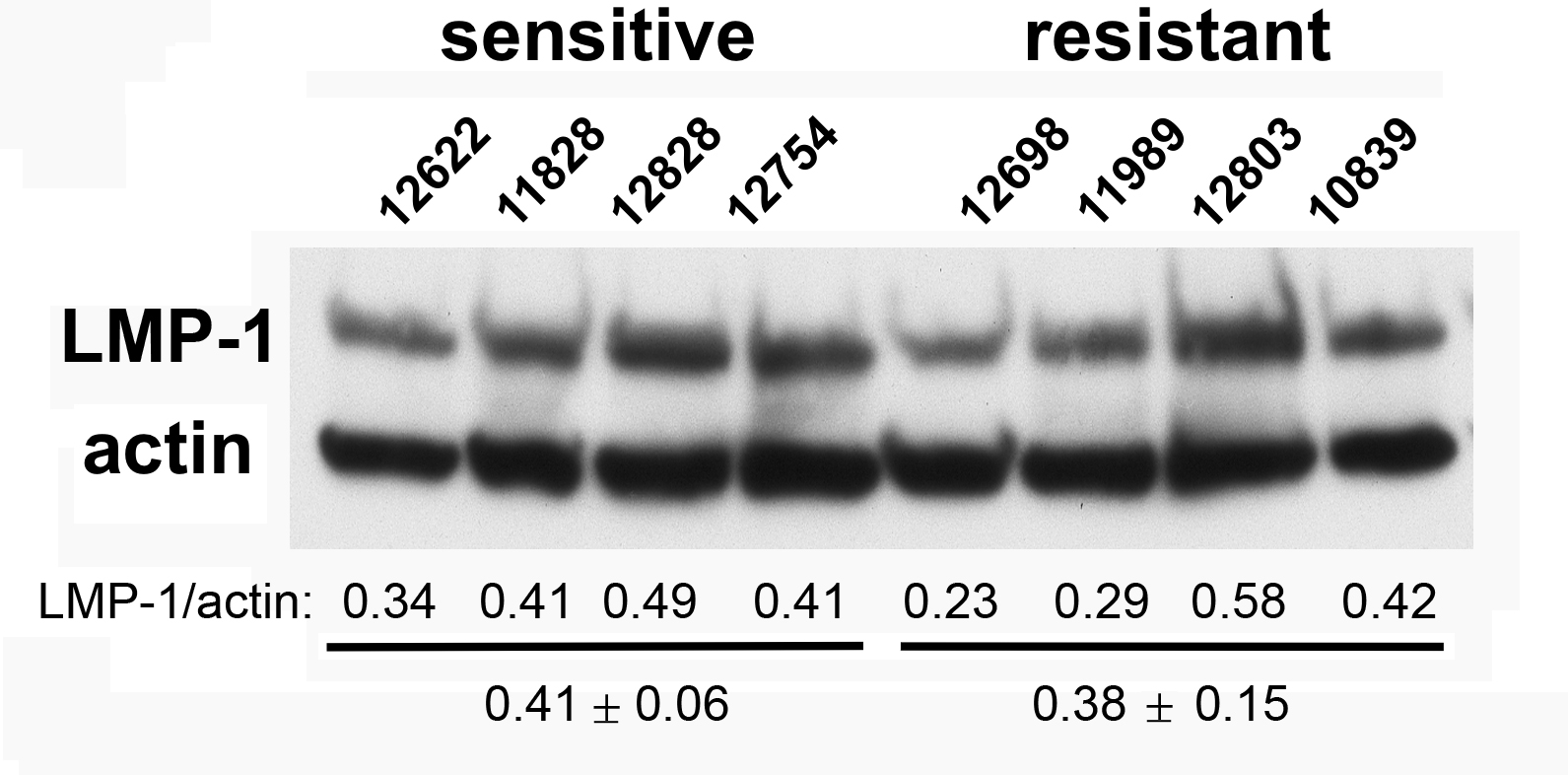

Supplement: Supplementary Fig. 2 — Western blotting was performed on cellular extracts from lymphoblastoid cell lines previously identified in surveys of cell lines as either sensitive (lanes 1-4) or resistant (lanes 5-8) toward rituximab. Following densitometry, relative LMP-1 protein levels are expressed after correction for equal protein loading using b-actin as a load control. The average (SEM) is provided for the sensitive and resistant groups. [file peerj-01-31-s002.jpg]

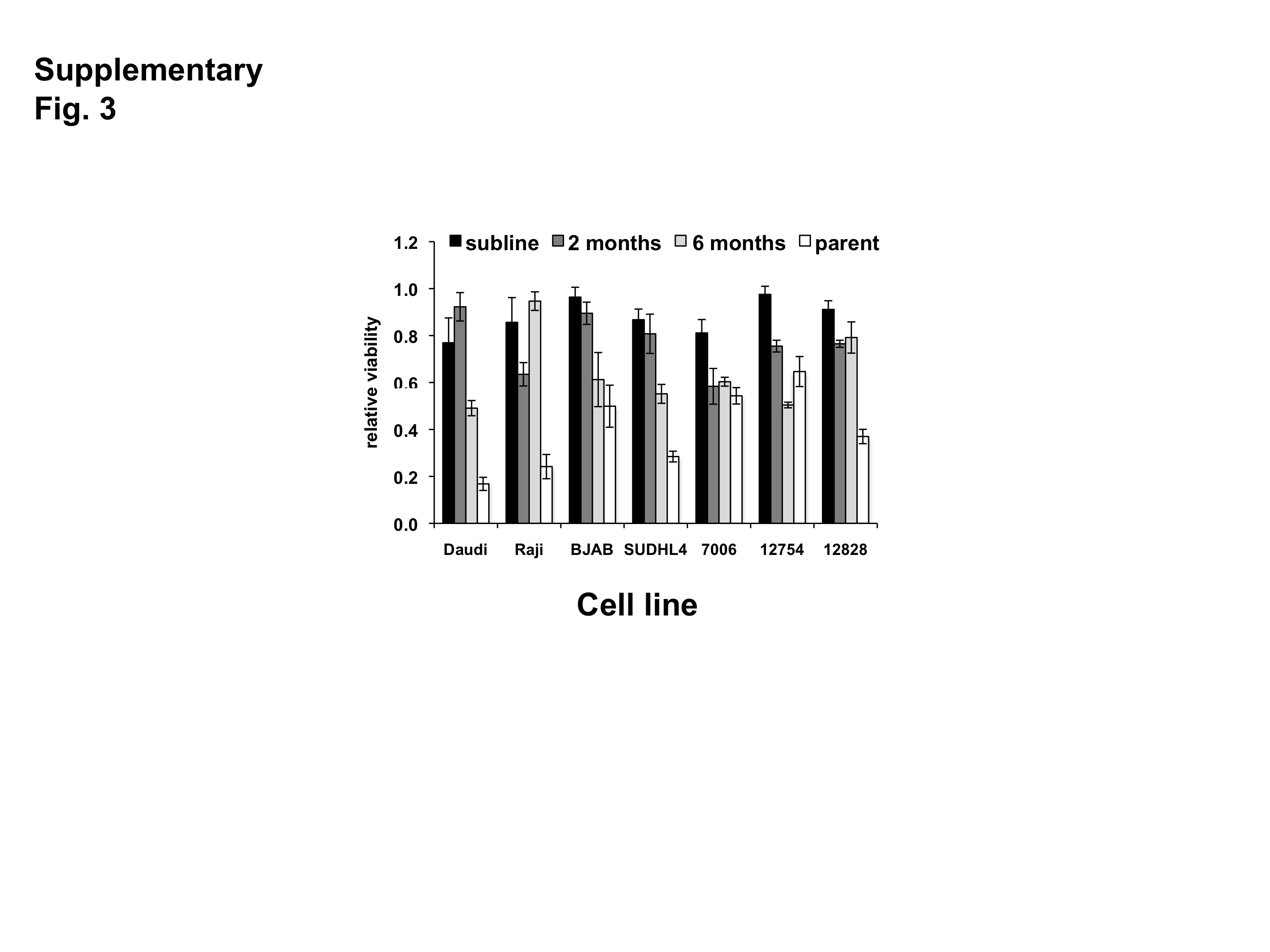

Supplement: Supplementary Fig. 3 — Resistant sublines were selected from parental cells as described in Methods. A) Viability assay comparing parental cell line and sublines toward rituximab in presence of human serum. Data are expressed as the average of duplicates (± SEM) and representative of three experiments. B) Viability assay comparing parental cell line, resistant subline, and resistant sublines removed from selective pressure for the indicated times. Data are expressed as the average of two experiments performed in duplicate (± SEM). [file peerj-01-31-s003.jpg]

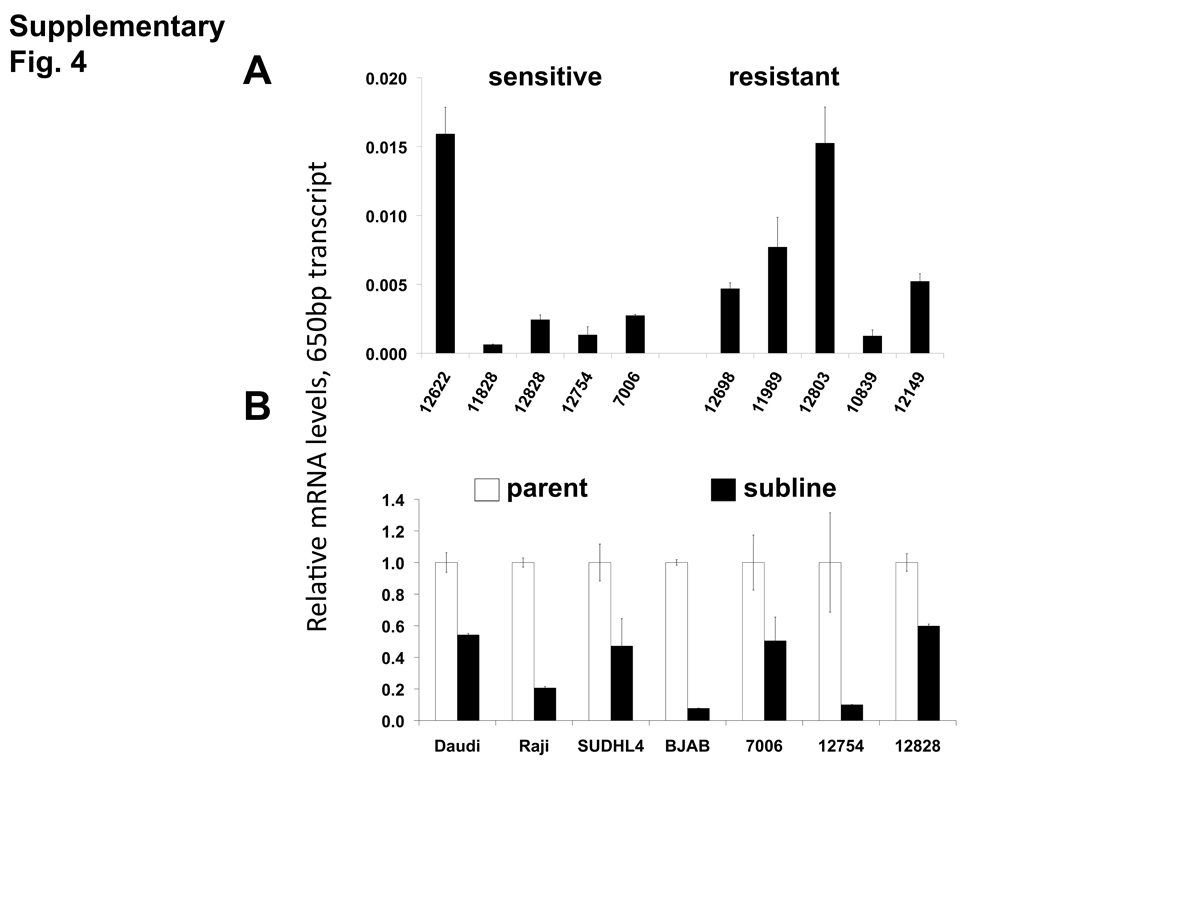

Supplement: Supplementary Fig. 4 — Levels of the 650 bp CD20 mRNA isoform (variant 1) measured by qPCR are shown in (A) lymphoblastoid lines that were identified as either relatively sensitive or resistant to rituximab, and (B) parental and resistant sublines. In (A), ratios are relative to the level of full-length CD20 of the lymphoblastoid line, 12622. In (B) resistant sublines are each normalized to the level of 650-bp transcript levels of the corresponding parental line (set to 1). Ratios of the 650 bp transcript to full-length CD20 ranged from 0.0006 to 0.016 in the lymphoblastoid cell lines and from 0.008 to 0.174 in the lymphoma cell lines. Results are the average of duplicates (± SEM). [file peerj-01-31-s004.jpg]
