# Supplementary material for: Analysis of innate and acquired resistance to anti-CD20 antibodies in malignant and nonmalignant B cells
Source: PeerJ. 2013 Feb 12;1:e31. doi: 10.7717/peerj.31 (PMC3628892; doi:10.7717/peerj.31)
Supplement: Supplementary Table 1 [file peerj-01-31-s005.docx]

**Supplemental Table 1: List of cell lines used in anti-CD20 study**

Lymphoblastoid (CEPH) lines

GM-:

6991 7006 7008 7010 7012 7019 7020 7021 7027 7040 7044 7045 7048 7062 7345 7342 7348 10838 10839 10847 10860 11820 11822 11824 11826 11827 11828 11983 11984 11985 11988 11989 11993 11997 11998 12000 12001 12002 12007 12141 12148 12149 12150 12151 12157 12238 12616 12617 12618 12619 12620 12698 12699 12701 12702 12704 12706 12752 12753 12754 12755 12756 12757 12758 12759 12764 12765 12766 12768 12769 12771 12772 12773 12801 12754 12802 12803 12804 12805 12806 12808 12809 12810 12824 12828 12848 12849 12867 12870 12871 12876 12883

Lymphoma lines

Daudi, Raji, BJAB, SUDHL4, SUDHL10, HT, Farage, DB, Jurkat
